# Measuring and Correction Methods of H.-J. Haase Improve Binocular Vision in Patients with Severe Anisometropia †

**DOI:** 10.3390/jcm14186367

**Published:** 2025-09-09

**Authors:** Shun-Huan Wang, Ya-Yu Chen, Mei-Lan Ko

**Affiliations:** 1 Department of Biomedical Engineering and Environmental Sciences, National Tsing Hua University, Hsinchu City 30013, Taiwan; 2 Department of Optometry, Mackay Medical College, New Taipei City 25245, Taiwan; 3 Department of Ophthalmology, National Taiwan University Hospital, Hsinchu Branch, Hsinchu City 302058, Taiwan

**Keywords:** heterophoria, stereo vision, fixation disparity, Measuring and Correction Methods of H.-J. Haase, anisometropia

## Abstract

**Background/Objectives:** To compare binocular vision status after spectacle correction using Measuring and Correction Methods of H.-J. Haase (MCH) and Optometric Extension Program (OEP) methods in patients with severe anisometropia and impaired fusion, including those with a history of monocular cataract surgery. **Methods:** This retrospective, observational comparative study analyzed the medical records of 34 patients with severe anisometropia (≥3.00 D) who were fitted with spectacles at Yi-Ming Optometry Clinic between January 2019 and May 2023. Patients were aged 32 to 82 years and had no ocular or systemic comorbidities affecting visual function. Binocular vision data previously recorded using the MCH and OEP methods were extracted and analyzed. All data are expressed as mean ± standard error, and *p*-values ≤ 0.05 were considered statistically significant. **Results:** The mean anisometropia and heterophoria measurements were 5.51 ± 0.45 and 2.26 ± 0.32 D (∆), respectively. The mean MCH and OEP oculus uterque visual acuity measurements were −0.020 ± 0.010 and 0.040 ± 0.010 log-MAR (*p* < 0.001), respectively. In the right eye, the horizontal prisms for MCH and OEP were 0.780 ± 0.128 and 0.020 ± 0.020 (∆) (*p* < 0.001), whereas those of the left eye were 0.790 ± 0.119 and 0.040 ± 0.025 ∆ (*p* < 0.001), respectively. The mean MCH and OEP stereo vision measurements were 97.560 ± 7.888 and 167.120 ± 17.295 arcsec (*p* < 0.001), respectively. **Conclusions:** The findings indicate that MCH prism resulted in superior stereo vision and binocular visual acuity compared to OEP in severe anisometropia patients.

## 1. Introduction

Anisometropia reduces depth perception and stereoacuity in binocular vision [1]. The estimated prevalence is 0.1% in the general population [2]. Approximately 20% of individuals exhibit an interocular refractive difference greater than 0.5D, with 2–3% having a difference of 3 D or more [3]. This anisometropia can induce aniseikonia, where individuals typically tolerate an image size difference of only up to 7% within 3 D of anisometropia [4,5].

Generally, anisometropia correction with spectacles is often not as optimal as with contact lenses, because spectacle lenses can induce differential image magnification, leading to different image sizes on the retinas compared to contact lenses [6]. Contact lens use and refractive surgery may result in the formation of clear retinal images, and they are used to treat anisometropia [7,8]; however, they have associated risks. Following cataract surgery in one eye, patients with high myopia may also experience the inconvenience and symptoms of anisometropia [9,10] if the difference in refractive power is ≥3D [11].

In cases with anisometropia, when contact lenses or surgery are contraindicated or declined, spectacle correction incorporating prisms may be considered as an alternative [10,12]. However, prescribing an appropriate spectacle correction with prisms can be challenging, and there is limited research on its effectiveness for severe anisometropia. Currently, two principal methods are available to guide prism prescriptions: the Measuring and Correction Methods of H.-J. Haase (MCH) [13,14,15], which is more commonly used in Central Europe [16,17], and the Optometric Extension Program (OEP) method [18,19,20], which is frequently applied in North America [16]. Despite their use in clinical practice, there is limited high-quality evidence available regarding how these methods influence final spectacle prescriptions in patients with severe anisometropia.

Previous studies exploring prism-based correction for anisometropia and aniseikonia using the MCH method have reported improvements in binocular vision function; however, these findings were based on extremely small samples, often involving only one or two participants [16,21]. Another small-sample study (*n* = 14) using video oculography showed some promise for prism correction, modestly improving the speed of one of three saccadic activities while slightly impairing smooth pursuits. The study also compared prisms and isekonic lenses. Isekonic lenses are designed to adjust the size of the retinal image without altering the refractive power by modifying factors such as lens thickness, base curve, and vertex distance to minimize image size differences. No statistically significant differences were found in other visual performance tasks, and the authors concluded that prism correction was easier to prescribe and fabricate than isekonic lenses, with no obvious disadvantages [22]. A larger survey conducted across 36 private practices in Germany and Switzerland (*n* = 857) reported that MCH-prescribed prisms led to substantial subjective improvements in symptoms, including visual acuity, photophobia, headaches, attention, fine and gross motor skills, and near-work discomfort, compared to prior spectacles with or without prisms. However, as this is currently the only large-scale study available, its generalizability remains uncertain, and further research is needed to confirm these findings across broader populations [17].

Conversely, some authors have questioned the reliability of MCH-based prescriptions. Measurement of fixation disparity with the MCH system has been described as unreliable [23], and no clear improvement in stereoacuity has been demonstrated [24,25]. This may be partly due to the lack of appropriate trial frames that better approximate natural viewing conditions. Furthermore, these critical studies also had small sample sizes, typically fewer than 10 participants, further limiting generalizability.

The objective of this study was to evaluate the corrective effects of spectacle prescriptions in severe anisometropic patients using the MCH and OEP methods, and to compare the differences between both prescription approaches.

## 2. Materials and Methods

### 2.1. Inclusion and Exclusion Criteria

In this retrospective, observational comparative study, the medical records of 60 patients with anisometropia, defined as an interocular refractive difference of ≥3.00 D, who visited Yi-Ming Optometry Clinic for spectacle correction between January 2019 and May 2023, were reviewed. Of these, 26 patients were excluded due to incomplete clinical data, a history of ocular or systemic conditions affecting visual function, concurrent ocular diseases, or other factors that rendered binocular vision assessment incomplete. The remaining 34 patients, aged between 32 and 82 years, included individuals who had previously undergone monocular cataract surgery, as well as those with uncorrected high anisometropia identified during spectacle prescription. All patients were managed with spectacle correction. All procedures were conducted in accordance with the Declaration of Helsinki and were approved by the Research Ethics Committee of National Tsing Hua University (approval number: 11204HM039), with full compliance with relevant local regulations.

### 2.2. Primary Outcome

The primary outcomes of this study were binocular visual acuity and stereopsis, which were compared between the MCH and OEP methods. These measures were selected because they directly reflect the functional impact of prism prescription on binocular vision performance in patients with severe anisometropia.

### 2.3. Equipment and Environment

Artificial illumination was provided to maintain consistent lighting throughout all assessments. Visual acuity measurements were conducted within an intensity range of 400–600 lux, which limits any potential effect on visual acuity to 0.012 logMAR [26]. All testing was performed under these controlled conditions to ensure reliability and comparability of results across participants.

Optometric infinity deviates considerably from real infinity because rays of light emanating from a point 6 m away from the eye possess a vergence of 0.17 D upon reaching the eye. A 0.33-D myope may achieve perfect focus and help read an additional line of letters in a test conducted at 3 m compared with that at 6 m. To minimize the effect of accommodation, it is preferable to maintain a test distance of 6 m [18].

**Figure 1:**
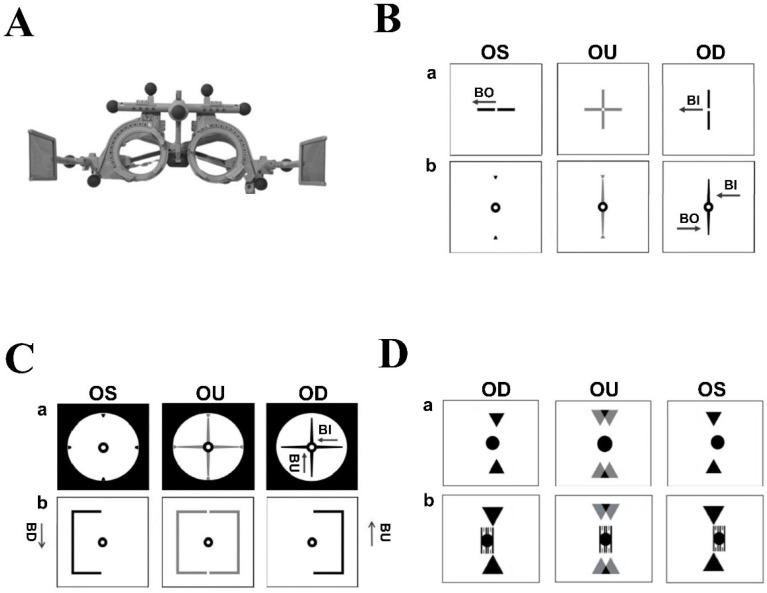
(**A**) Trial frame (OCULUS UB4 H-A): Adjusts the patient’s eye position for examination. (**B**) MCH Heterophoria measurement: The corresponding prism is added to align the graphics [13,14,15]. (**a**) Cross test, (**b**) Pointer test. (**C**) (**a**) Double pointer test, (**b**) Rectangle test. (**D**) (**a**) Stereo triangle test, (**b**) Stereo-balance test.

In Figure 1A, a trial frame with analyzers (polarizing filters) is crucial for conducting MCH testing, and the height of both sides of the trial frame should be independently adjusted [15]. Furthermore, the pantoscopic tilt angle on one side, vertex distance, height of each eye, interpupillary distance, and height difference between the ears can be adjusted to avoid generating a prismatic effect (P = d∙S) [27].

Lorgnette-type analyzers are not recommended to measure heterophoria, following the guidelines of the Internationale Vereinigung für Binokulares Sehen (IVBS). The primary drawbacks of a phoropter may induce unnatural head and body postures, particularly during near vision assessments, and the stimulus to proximal accommodation [28,29]. Additionally, the changing visual points resulting from head and body movements can produce a prismatic effect [27]. Moreover, phoropters do not allow patients to move around while wearing them.

### 2.4. Procedures

All participants were examined by the same optometrist using identical instruments under uniform conditions. Thirty-four patients with anisometropia ≥ 3.00 D were recruited and examined using the MCH and OEP methods. Both methods involve the same procedures from objective refraction to binocular balance, except for heterophoria measurement. First, objective refraction was performed using an autorefractor (i.Profiler (Plus); Carl Zeiss Vision, Aalen, Germany) to initially determine the spherical and astigmatism powers. Subsequently, a trial frame (OCULUS UB4 H-A; OCULUS, Wetzlar, Germany) was used to set the patient’s pupil distance, eye position height, and vertex distance (Figure 1A). The subjective correction aimed at maximum visual acuity was then conducted by using the Snellen chart positioned 6 m away on a liquid crystal display (Zeiss Visuscreen 500; Carl Zeiss Vision, Aalen, Germany). The relating process included covering one eye (OS), obtaining the initial refractive power with the autorefractor, adding +1.50 D spherical power, and then incrementally increasing minus power until no further improvement in vision was observed. These steps were repeated for the other eye. Thereafter, the bichrome test was employed to refine the subjective correction; if the letters on the red side stood out more, minus power was added; if the letters on the green side stood out more, plus power was added until the two background letters appeared the same. Finally, the Jackson cross cylinder (Jackson cross cylinder; OCULUS, Wetzlar, Germany) was manipulated until optimal alignment of axis and cylinder was achieved.

In the MCH heterophoria measurement, a structured series of tests is performed to assess fixation disparity. Polarizing filters are placed in front of both eyes, and the following tests are then conducted:

1. Cross test: Alignment between a vertical line (right eye) and a horizontal line (left eye) was assessed. Prisms (base in/out) were adjusted until the cross aligned (Figure 1B(a)).
2. Pointer test: A vertical pointer (right eye) was aligned with clock markings (left eye). Deviations were corrected with prism adjustments until alignment was achieved (Figure 1B(b)).
3. Double pointer test: Alignment of vertical and horizontal pointers with clock markings was checked. Deviations (horizontal or vertical) were corrected with corresponding prisms until the crosshair aligned with the marking (Figure 1C(a)).
4. Rectangle test: Right- and left-eye rectangles were aligned. Vertical misalignments were corrected using base-up or base-down prisms until the rectangles coincided (Figure 1C(b)).
5. Stereo triangle test: Assessed fixation disparity by comparing clarity and speed of crossed vs. uncrossed disparity. Appropriate prisms were applied until moving triangles appeared centered and aligned with the calibration line (Figure 1D(a)) [14,15].
6. Stereo-balance test: Detected lateral displacement of triangles under stereo disparity. Deviations were corrected with prisms until triangles aligned with the central scale (Figure 1D(b)). After the heterophoria measurement, the next step was binocular balance testing.

Detailed descriptions of the MCH heterophoria measurement are provided in the Supplementary Materials.

### 2.5. The Measurement of Heterophoria by the OEP Method

The OEP method employed the Von Graefe test to measure heterophoria and determine prism correction using Sheard’s or Percival’s criterion [30,31]. The procedure was as follows:

1. Setup: A prism dissociation of 6Δ base-up was placed in front of the right eye, while a 12Δ base-in measuring prism was placed in front of the left eye using a phoropter.
2. Fixation target: Participants focused on a 20/30 visual acuity letter line at both near and distance while maintaining clarity.
3. Alignment task: They were instructed to shift gaze to a lower target and indicate when the upper target aligned just above the lower one.
4. Adjustment: Horizontal (12Δ) and vertical (6Δ) prisms were adjusted in one-diopter increments until alignment was reported.
5. Repetition and averaging: The procedure was repeated three times, and average values were recorded.
6. Criterion application: Sheard’s criterion was applied for exophoria cases, while Percival’s criterion was applied for esophoria cases.

The MCH approach was intuitively applied to examine both eyes using the cross, pointer, double pointer, rectangle, stereo triangle, and stereo-balance tests. The prism measured in MCH was directly incorporated into the prescription, whereas the OEP method applied Sheard’s or Percival’s criterion to determine the prism prescription. Other aspects of the examinations were identical between the two methods. After the measurement of heterophoria, both OEP and MCH prescriptions were evaluated using the Cowen test, in which a polarizing filter was placed in front of both eyes, and either minus or plus power lenses were incrementally added until the two background rings were perceived as equal. The final step was to compare and analyze the recorded visual acuity and stereoacuity of the OEP and MCH prescriptions. Stereoacuity was assessed using the graded circle test, which can measure stereoacuities from 400 to 20 arcsec (Vision Assessment Corporation, Elk Grove Village, IL, USA).

### 2.6. Statistical Analyses

Statistical Package for the Social Sciences (SPSS) version 25.0 (IBM Corp., Armonk, NY, USA) was used for statistical analyses of spherical and astigmatism powers, astigmatism axes, horizontal and vertical prism diopter, vergence, stereo vision, and visual acuity. A nonparametric Wilcoxon signed-rank test was performed to compare continuous variables, as the test does not assume normality of the data distribution. All data are expressed as mean ± standard error. Descriptive statistics such as percentage, mean, and standard error were employed. Results with *p* ≤ 0.05 were considered statistically significant.

## 3. Results

**Figure 2:**
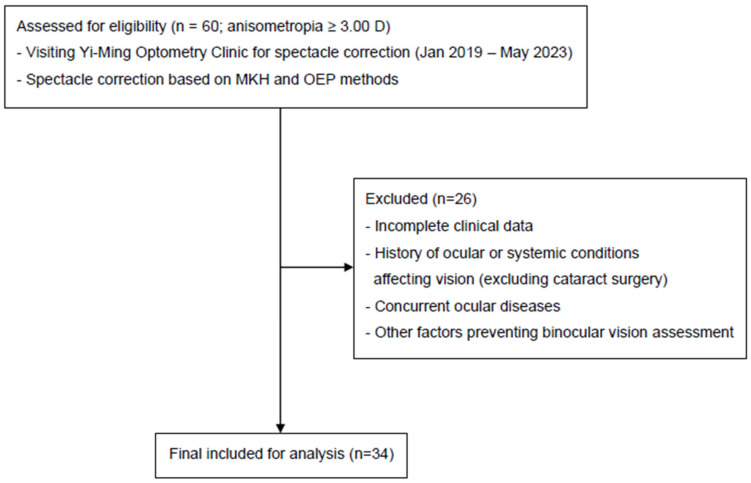
Strengthening the Reporting of Observational Studies flow diagram showing participant screening, eligibility, and inclusion in the study.

Thirty-four (sixteen male and eighteen female) participants were included. Their mean age was 48.32 ± 2.05 years. The flow of patient selection and inclusion is shown in Figure 2. The mean right eye spherical was −4.62 ± 0.91 D, cylinder was −0.89 ± 0.11 D, and spherical equivalent was −5.06 ± 0.91 D; the mean left eye spherical was −2.73 ± 0.69 D, cylinder was −1.18 ± 0.16 D, and spherical equivalent was −3.31 ± 0.71 D. The mean anisometropia was 5.51 ± 0.45 D and mean heterophoria was 2.26 ± 0.32 D (∆). The mean right eye visual acuity was 0.02 ± 0.01 logMAR, and the left eye visual acuity was 0.11 ± 0.06 logMAR (Table 1).

Specifically, the mean MCH oculus uterque visual acuity was −0.020 ± 0.010 logMAR, and that for the OEP method was 0.040 ± 0.010 logMAR (*p* < 0.001). The horizontal MCH prism in the right eye was 0.780 ± 0.128 (∆), and that for the OEP method was 0.020 ± 0.020 (∆) (*p* < 0.001). The horizontal MCH prism of the left eye was 0.790 ± 0.119 (∆), and that for the OEP method was 0.040 ± 0.025 (∆) (*p* < 0.001). The MCH stereo vision was 97.560 ± 7.888 (arcsec), and that for the OEP method was 167.120 ± 17.295 (arcsec) (*p* < 0.001) (Table 2). Other parameters did not show significant differences. There were statistically significant differences between the MCH and OEP methods in oculus uterque visual acuity, oculus dexter horizontal prism, oculus sinister horizontal prism, and stereo vision; however, there was no significance in mean horizontal prism.

## 4. Discussion

Generally, the visual centers of patients can only tolerate anisometropia with a power difference of within 3 D [32]. Here, the MCH prescription successfully enabled tolerance to a maximum anisometropia of 14.46 D, with an average of 5.51 ± 0.45 D without double vision. The MCH and OEP stereo visions were 97.560 ± 7.888 and 167.120 ± 17.295 arcsec, respectively. The mean oculus uterque visual acuity of MCH and OEP were −0.020 ± 0.010 and 0.040 ± 0.010 logMAR, respectively. These findings showed the MCH prescription yields better results in both binocular vision and stereopsis. As an illustrative case, the maximum anisometropia occurred in a 48-year-old male patient who developed unilateral high myopia after cataract surgery, resulting in extreme anisometropia (right eye −14.00 D with −0.38 D cylinder, SE −14.19 D; left eye +0.77 D with −1.00 D cylinder, SE +0.27 D). Using the MCH-based prescription, this case achieved successful binocular tolerance without diplopia. This remarkable tolerance to high anisometropia may be explained by the patient’s acquired condition (post-cataract surgery monocular pseudophakia), which might have facilitated better neural adaptation compared to developmental anisometropia.

We investigated whether the MCH method showed better visual performance. The MCH method and OEP method both involve the use of a trial frame (OCULUS UB4 H-A) (Figure 1A), which eliminates unnatural postures caused by comprehensive refraction and avoids the use of false prisms [27]. Both distance is set at a minimum of 5 m to prevent accommodative stimuli [18]. The light level is maintained at 400–600 lx during refraction for stable visual acuity assessment [26]. This is essential for correcting fixation disparity and improving oculus uterque visual acuity and stereopsis in patients with anisometropia after correction. One notable difference between the MCH- and OEP-based prescriptions was in the horizontal prism. According to the guidelines, both the MCH and OEP methods fully correct vertical prism, which explains why no significant differences were observed in the vertical component between the two methods.

The enhanced binocular vision and stereopsis achieved with the MCH prescription were suspected to have used prisms, which assist in precise foveal alignment in both eyes, thus facilitating the highest visual acuity when the image is centered in the field of view [33]. The fovea, which spans approximately 1° of visual angle on either side of the fixation point, contains the highest density of cones in the retina. However, beyond this central region, cone density declines rapidly with increasing retinal eccentricity (ε), resulting in a marked reduction in visual acuity [34,35]. According to the definition of prism diopter (Δ), one prism diopter can cause light to deviate by 10 mm at a distance of 1000 mm. Assuming that the axial length of the eye is 25 mm, a deviation of four prism diopters corresponds to approximately a 1 mm displacement of the visual axis, equivalent to about 2.28°, away from the fovea. Such a displacement may negatively affect visual acuity [36]. For patients experiencing convergence insufficiency type exophoria, four-prism diopters of base-in prisms can improve binocular vision [37]. The effects of prisms prescribed via the MCH method have been compared to those determined during standard orthoptic examinations [38]. In a cohort of asthenopic patients, MCH-prescribed prisms demonstrated sustained improvements in relative accommodation and vergence measures for up to five years. Although changes in accommodative power over time may necessitate updates to the prescription [39,40], clinical vergence assessments and contrast sensitivity tests [41] have consistently highlighted the positive impact of these prisms.

During binocular fixation on the same point, each point on the retina of one eye corresponds to a specific point or area (Panum’s area) on the retina of the other eye. This correspondence allows the brain to fuse the images focused on each retina into a single image. Ophthalmic prisms align the patient’s visual axes within Panum’s fusional area, positioning them on the horopter to enhance binocular fusion [42]. The misalignment of visual axes during binocular vision leads to diplopia, as the retinal images cannot merge into a single image owing to the absence of point-to-point or point-to-area correspondence [42]. The Panum’s area enables a certain level of imprecision in binocular fixation without causing diplopia by permitting slight deviations of the eyes from the fixation point, where the visual axes do not fall precisely on the corresponding retinal points but are within related Panum’s areas. This minor deviation from the fixation point, without diplopia, is referred to as fixation disparity [43]. The associated phoria is closely related to fixation disparity. Non-optimal binocular coordination is a relatively common issue, characterized by considerable individual differences and impairments that can manifest as reduced stereo vision, weakened vergence functions, decompensated heterophoria, or complaints related to asthenopia [30,44]. Eliminating fixation disparity involves using sphere or prism power [45,46]. Hans-Joachim Haase reported that prolonged fixation disparity could result in disparate correspondence between the central areas of both retinas, leading to impaired stereoacuity. It was also noted that prismatic spectacles could help restore bicentral fixation, ultimately enhancing stereoacuity [14]. This could potentially account for the superior oculus uterque visual acuity and stereopsis with MCH-based prescriptions observed in severe anisometropic patients.

However, this study still had a few limitations. Two patients with mild amblyopia were included: one with 20/32 in the right eye and 20/20 in the left eye, and another with 20/32 in both eyes. After full correction, both achieved stereopsis of 100–200 arcsec. As these patients had not previously received full correction, full correction would have resulted in diplopia. Between the two methods, significant differences were observed. The MCH method demonstrated superior outcomes in binocular visual acuity and stereopsis compared to the OEP method, without inducing diplopia. Among the study population, five patients had hyperopic anisometropia. Both the MCH and OEP methods demonstrated significant differences in outcomes for these patients, consistent with findings in myopic patients. The cylinder values of participants ranged from 0 to −3.25 D, and significant differences between methods were observed across this range, indicating that refractive error type or astigmatism degree did not hinder the effectiveness of prism correction. We also explored whether higher anisometropia correlated with larger phoria values, but no clear relationship was found. Various methods for assessing heterophoria exist, and in this study, we only compared the outcomes of MCH and OEP methods for patients with severe anisometropia. Determining the superiority of an optometric method and discussing its universal applicability requires further experimentation with a larger sample, especially given the extreme nature of participants’ prescriptions. While additional subgroup analyses were not applicable in this study, future studies could further investigate potential differences across refractive error type, degree of cylinder, and amblyopia severity. If the equipment setup and the trial frame are not provided according to the IVBS guidelines, the refraction and fitting of prisms for anisometropia using the MCH method will not be successful. Due to the variability in each patient’s accommodative convergence/accommodation (AC/A) ratio, the prism diopters used are not directly related to the magnitude of anisometropia. In our clinical practice, no significant improvement in visual performance was observed in anisometropic patients with an interocular refractive difference of less than 3.00 diopters (unpublished data). When assessed using the MCH and OEP methods, no differences were found in binocular visual acuity and stereopsis, and these patients did not report any binocular vision-related complaints. This aligns with the findings of Miriam Kromeier et al. [25], who demonstrated that for individuals with good stereopsis and low levels of anisometropia, the presence or absence of prisms does not affect stereoscopic vision. The MCH prescription may be more relevant for individuals with anisometropia, while the OEP method proved to be relatively faster and more convenient. In conclusion, the MCH prescription significantly enhances binocular vision and stereopsis in patients with high anisometropia by using prisms, ensuring precise foveal alignment in both eyes and facilitating optimal visual acuity. The positive effect of using these prisms was evident in the findings of clinical vergence assessments [39,40] and contrast sensitivity tests [41], as shown in our study, which showed that the MCH method provides superior stereopsis and oculus uterque visual acuity. Ultimately, these insights hold promise for improving the quality of life and visual outcomes for people suffering from these conditions.

## 5. Conclusions

In patients with severe anisometropia and impaired binocular fusion, the MCH method demonstrated superior outcomes in binocular visual acuity and stereopsis compared to the OEP method. This improvement is likely attributed to the precise prismatic correction used in MCH, which aligns the visual axes within Panum’s fusional area and reduces fixation disparity. While both methods utilize similar refractive procedures and conditions, the MCH approach allowed patients to tolerate greater degrees of anisometropia without experiencing diplopia. The findings suggest that MCH-based prescriptions may be especially beneficial for patients with high anisometropia, though further large-scale studies are needed to confirm these results and establish broader clinical guidelines.

## Figures and Tables

**Table 1:** Demographics and ocular data for patients with anisometropic eyes.

Number = 34	Mean ± SE (Min, Max)
Age (years)	48.32 ± 2.05 (min 32, max 82)
Sex	
Male	16 (47%)
Female	18 (53%)
Sph/Cyl/SE	
OD	−4.62 ± 0.91 (min −14.00, max 5.12)/−0.89 ± 0.11 (min −3.25, max 0.00)/−5.06 ± 0.91 (min −14.19, max 4.69)
OS	−2.73 ± 0.69 (min −16.50, max 5.25)/−1.18 ± 0.16 (min −3.00, max 0.00)/−3.31 ± 0.71 (min −16.87, max 5.00)
Anisometropia (SE)	|5.51 ± 0.45| (min 3.00, max 14.46)
Phoria (▵)	2.26 ± 0.32 (min 0.50, max 8.75)
VA (logMAR)	
OD	0.02 ± 0.01 (min −0.10, max 0.20)
OS	0.11 ± 0.06 (min 0.00, max 0.20)

Abbreviations: Sph, spherical; Cyl, cylinder; OD, oculus dexter; OS, oculus sinister; SE, spherical equivalent; VA, visual acuity; (▵), prism; min, minimum; max, maximum. Note: Data are presented as mean ± standard error.

**Table 2:** Multivariate analysis of Optometric Extension Program (OEP) and Measuring and Correction Methods of H.-J. Haase (MCH) groups.

	MCH	OEP	*p*
	Mean ± SE (Min, Max)	Mean ± SE (Min, Max)	
OUVA (logMAR)	−0.020 ± 0.010 (min −0.079, max 0.097)	0.040 ± 0.010 (min 0.201, max 0.000)	<0.001
ODH (▵)	0.780 ± 0.128 (min 0.000, max 2.750)	0.020 ± 0.020 (min 0.000, max 0.688)	<0.001
OSH (▵)	0.790 ± 0.119 (min 0.000, max 3.000)	0.040 ± 0.025 (min 0.000, max 0.688)	<0.001
ODV (▵)	0.260 ± 0.093 (min 0.000, max 2.450)	0.350 ± 0.131 (min 0.000, max 3.500)	0.129
OSV (▵)	0.420 ± 0.177 (min 0.000, max 5.000)	0.320 ± 0.132 (min 0.000, max 3.500)	0.203
SV (‘’)	97.560 ± 7.888 (min 40.000, max 200.000)	167.120 ± 17.295 (min 50.000, max 400.000)	<0.001

Abbreviations: OUVA, oculus uterque visual acuity; OD, oculus dexter; OS, oculus sinister; H, horizontal; V, vertical; SV, stereo vision; (‘’), arc second; (▵), prism; min, minimum; max, maximum. Note: Data are presented as mean ± standard error. The *p*-value in the table is from values compared among different method groups.

## Data Availability

Data are contained within the article.

## Supplementary Materials

The following supporting information can be downloaded at https://www.mdpi.com/article/10.3390/jcm14186367/s1, MCH Heterophoria Measurement.
