# Supplementary material for: Measuring and Correction Methods of H.-J. Haase Improve Binocular Vision in Patients with Severe Anisometropia
Source: J Clin Med. 2025 Sep 9;14(18):6367. doi: 10.3390/jcm14186367 (PMC12470504; doi:10.3390/jcm14186367)
Supplement: Supplementary file 1 [file jcm-14-06367-s001.zip › jcm-3810589-supplementary.pdf]

Supplementary Methods: MCH Heterophoria Measurement

Cross test: the individual's perception of a vertical line with the right eye and a horizontal line with the left eye was verified. Alignment of the crosshair reticle was scrutinized, with prism adjustments made based on the individual's indications of deviation, employing either base out or base in prisms as appropriate. For example, if an individual indicated that the vertical line shifted to the right, a prism with base out was added; the same procedure was followed for the other side. The examination was complete when the cross aligned (Figure 1 B(a)). A frame displayed to both eyes serves as a fusional lock, while the alignment of vertical and horizontal lines for both eyes is used to measure fixation disparity.

Pointer test: assessed the alignment between a vertical pointer seen by the right eye and clock markings perceived by the left eye. Prism adjustments were implemented in response to indications of deviation, facilitating precise alignment. This central (central circle)-plus-peripheral (vertical line) fusional lock uses clock markings and a vertical pointer to measure fixation disparity. For example, if an individual indicated that the vertical pointer shifted to the right, a prism with base out was added; the same procedure was followed for the other side. The examination was complete when the pointer lined up with the marking (Figure 1 B(b)).

Double pointer test: conducted to ensure alignment between a crosshair pointer perceived by the right eye and clock markings viewed by the left eye. Adjustments were made according to the individual's indications of deviation. If the horizontal pointer shifted upwards or

downwards, prism adjustments were executed to rectify the misalignment, employing base up or base down prisms accordingly. For example, if an individual indicated that the double pointer shifted to the left, a prism with base in was added; the same procedure was followed for the other side. This central (central circle) plus peripheral (vertical and horizontal lines) fusional lock uses clock markings and vertical-horizontal pointers to measure fixation disparity. The examination was complete when the crosshair lined up with the marking (Figure 1 C(a)).

Rectangle test: evaluated the alignment between right- and left-side rectangles perceived by each eye. Prism adjustments were carried out to address any observed deviations, ensuring alignment of the two rectangles. For example, if an individual indicated that the right-side rectangle shifted upwards, a prism with base up was added to the right eye or prism with base down was added to the left eye; the same procedure was followed for the other side. This central (central circle) plus peripheral (rectangle) fusional lock measures fixation disparity through the height difference between the two rectangles. The examination was complete once the two rectangles aligned (Figure 1 C(b)).

Stereo triangle test: focused on the promptness and clarity of stereo percept between crossed and uncrossed disparity to evaluate fixation disparity. This central (central circle) plus peripheral (triangle) fusional lock measures fixation disparity by assessing whether the moving triangles have the same speed and remain centered. If triangles are perceived more promptly

and distinctly in front of the fixation point (crossed stereo disparity) than those behind it (uncrossed stereo disparity), it indicates eso fixation disparity. This inference was based on the rationale that in eso fixation disparity, triangles with crossed stereo disparity will appear closer to the foveal center of the deviating eye, whereas in exo fixation disparity, triangles with uncrossed stereo disparity will be closer to the foveal center. [14, 15] When the corresponding prism was added and the upper and lower triangles aligned with the center of the calibration line, the examination was complete (Figure 1 D(a)).

Stereo-balance test: designed to detect any lateral displacement of triangles under crossed or uncrossed stereo disparity conditions. This central (central circle) plus peripheral (triangle) fusional lock measures fixation disparity by checking whether the moving triangles align with the central scale. An individual was required to indicate the direction of displacement, which was measured relative to scales positioned above and below the fixation point. Specifically, if the triangles were presented with crossed stereo disparity, triangles shifting to the right indicated exo fixation disparity. When the corresponding prism was added and the upper and lower triangles aligned with the center circle, the examination was complete (Figure 1 D(b)). After the heterophoria measurement, the next step was binocular balance testing.

Cowen test: Involved placing a polarizing filter was positioned in front of both eyes, and either minus or plus power was incrementally added until the two background rings were perceived as the same.
